# Mobile phone usage in patients with type II diabetes and their intention to use it for self-management: a cross-sectional study in Iran

**DOI:** 10.1186/s12911-020-1038-y

**Published:** 2020-02-07

**Authors:** Fatemeh Rangraz Jeddi, Ehsan Nabovati, Rahele Hamidi, Reihane Sharif

**Affiliations:** 1 Health Information Management Research Center, School of Allied Health Professions, Kashan University of Medical Sciences, Pezeshk Blvd, 5th of Qotbe Ravandi Blvd-Pardis Daneshgah, Kashan, 8715973449 Iran; 2 Department of Health Information Management & Technology, School of Allied Health Professions, Kashan University of Medical Sciences, Kashan, Iran; 3 Student research committee, Kashan University of Medical Sciences, Kashan, Iran

**Keywords:** Mobile health, Smartphone, Self-management, Type II diabetes

## Abstract

**Background:**

Mobile health has potential for promotion of self-management in patients with chronic diseases. This study was conducted to investigate smartphone usage in patients with type II diabetes and their intention to use it for self-management.

**Methods:**

This cross-sectional study was conducted in 2018 with 176 patients with type II diabetes visiting a specialized diabetes clinic or one of two endocrinology and metabolism specialists in north of Iran. Data were collected using a validated questionnaire containing items on demographic characteristics, disease information, use of mobile phones, smartphones and the internet, and intention to use mobile phones for diabetes self-management.

**Results:**

The majority of the participants had mobile phones (94.9%), smartphones (61.1%), and daily access to the internet (81.3%), and used phones two hours per day on average (80.1%). They mostly used mobile phones to contact friends (89.2%) and search for information (50.6%), and their greatest intention for using smartphones and the internet for self-management was related to dietary planning (96%), checking blood glucose (90.9%), and contacting specialists (87.5%). Younger participants were more interested in using smartphone applications (apps) (*P* < 0.001). About half of the participants argued that using apps can be interesting (54%) and useful (50%) for diabetes management, and intended to use apps much more in future (48.3%).

**Conclusions:**

The majority of patients with type II diabetes are inclined to use mobile phone and the Internet, especially to plan their diet, check blood glucose, and contact their doctors. The present study provides valuable information for designing and implementing interventions based on mHealth to promote self-management in type II diabetes.

## Background

Type II diabetes mellitus (T2DM), compromising the majority of patients with diabetes, has turned into a major health concern throughout the world [1]. According to the WHO estimates (2016), there are 422 million patients with diabetes over 18 years old worldwide [2], which will increase to 552 million patients by 2030 [3]. This increment is higher in developing countries than in developed countries [4]. A meta-analysis has shown that the prevalence of T2DM in Iran is higher than other developing countries [5]. The prevalence of diabetes in this country was reported about 10.3% in 2016 [6]. Diabetes is known as the seventh cause of death in the world [7] and has many complications including cardiovascular, cerebrovascular, peripheral vascular, retinopathy, nephropathy, diabetic foot, amputation, and depression [8]. Managing chronic diseases is challenging as diabetic patients require knowledge and skills in understanding the needs of medical care, and thus diabetes self-management is crucial, as part of a patient’s commitment to preventing disease complications [9].

Improving self-management behaviors is the first step toward helping patients with diabetes to better control their disease [10]. Successful management of diabetes depends on the individual’s ability to have effective self-management behaviors such as proper use of the prescribed medications, adherence with dietary and activity regimens, self-monitoring, and coping with the psychological impact of living with a chronic disease [11]. However, many patients with diabetes are faced with several barriers that interfere with self-management in achieving glycemic control, the body fat and weight, controlling blood pressure and blood fat, and having an optimizing diet [12, 13]. Given the high and increasing prevalence of diabetes and the importance of self-management, inexpensive and practicable self-management methods should be considered for these patients [14]. It has been shown that diabetes self-management can be improved with mobile phone interventions since they have potential to support therapy management, improve therapy adherence, and prevent disease complications [15].

Currently, mobile-based educational interventions are fairly new and can switch the focus of care and treatment from the clinic to the patient’s daily life [16]. Not only have these interventions improved clinical outcomes and self-management skills, but have also reduced the health-related costs and the frequency of the patient visits to the clinic [17–19]. Interventions based on information and communication technology including mobile health technology (mHealth), have a good potential for promoting self-management through behavior change supports (such as providing information, training, and reminders) [20, 21]. mHealth is a part of electronic health (eHealth), and implies using mobile phones and wireless technologies to improve health-related services [22]. Telemedicine tools (e.g. text messaging) and smartphone applications (apps) can facilitate and improve the patient-provider communication [23]. Ease of use and portability are the potential benefits of mHealth tools for prevention, diagnosis, and treatment of diseases, and also to enhance access to health services and reduce costs [24].

Despite its potential benefits and increasing interest in it, mHealth has not been implemented much in practice [25]. One of the key components for successful implementation of any mobile health intervention is its users’ positive attitude toward the use of this technology [26, 27]. According to the review of literature, several studies have already investigated the use of mHealth applications and the attitude of patients with diabetes on their use in developed countries [28–30]. In Canada, Dobson et al. (2015) investigated the current use and attitude of patients with T2DM on the use of the Internet and mobile phone apps in diabetes self-management, and reported that patients, especially younger ones, are more interested in adopting these technologies for their diabetes self-management [29]. In England, Humble et al. (2016) investigated the use and intention of patients with diabetes regarding mHealth technology for self-management and argued that younger patients use the Internet and apps more, and are interested in using apps for managing their diabetes [30]. A study conducted by Boyle et al. (2017) in New Zealand, only investigated the diabetes patients’ use of mobile phone apps and their features. Their results showed that 80.4% of respondents (56% with T1DM, 44% with T2DM) did not use diabetes apps [28]. To our knowledge, there is limited evidence on the use of mHealth applications and the attitude of patients with T2DM on their use in developing countries. In Iran, Jafari et al. (2015) only studied Internet access and Internet-related issues of patients with T2DM, and reported that patients had poor level of the Internet use and the Internet was not the main diabetes-related information source [31].

The number of smartphone apps relating to self-management of chronic diseases, especially diabetes, is increasing [32]. In contrast to developed countries such as the USA and England, in Iran as a developing country, no study has yet investigated the attitude and intention of patients with T2DM regarding the use of mobile phone capabilities (such as the Internet and apps) for self-management. Thus, the present study aimed to investigate the use of mobile phones in patients with T2DM and their intention to use them for self-management.

## Methods

### Study design and setting

The present cross-sectional study was conducted in spring 2018 in the city of Ardabil in northwestern Iran. Participants selected by convenience sampling method, included patients with T2DM visiting Imam Khomeini hospital’s diabetes clinic or one of two endocrinology and metabolism clinics. Patients with at least primary school education whose diabetes had been confirmed by a specialist qualified for participation. Patients unwilling to take part in the study were excluded. The required sample size was found 173 patients based on a similar study [29], and taking into account 93% daily use of the Internet in that study, 99% confidence, and maximum error of 0.05, and using n = (Z^2pq)/d^2^.

### Questionnaire

Data were collected using a questionnaire developed according to Dobson et al. study [29] and modified based on the study objectives, and views expressed by a health information management expert, a medical informatics expert, and an endocrinologist, who confirmed its face validity. Content validity of the questionnaire was assessed by 12 experts (in health information management, medical informatics, and endocrinology). The item content validity (i.e. relevance and clarity) plus comprehensiveness of the entire tool was assessed according to the views expressed by the mentioned experts in the form of Likert scale (from unfavorable = 1 to totally favorable = 4) [33]. Content validity was assessed for each item based on content validity index (CVI), such that items with CVI < 0.7 were revised and modified. Also, content validity ratio (CVR) was found according to Lawshe Table, such that items with CVR < 0.56 were eliminated. Reliability of the questionnaire was assessed by split-half and Cronbach’s alpha method (0.82).

After validation, the final questionnaire (Additional file 1) contained 30 items in five parts, including demographic details (5 items), disease information (5 items), the use of mobile phones and the Internet (6 items), intention to use mobile phone apps to control diabetes (11 items), and general explanations (3 items). In the demographic part, items were about gender, age, place of residence, education and occupation. In disease information part, participants were asked of their height, weight, duration of diabetes and other chronic diseases. They were also asked about diabetes management difficulties. These difficulties included choosing an optimizing diet, adequate physical activity, communication with the doctor and other healthcare providers, relationship with friends and family, blood glucose diary, and lack of clear and precise diabetes management goals. In the use of mobile phones and the Internet, participants were asked if they had a mobile phone and access to the Internet, and how many hours per day they used them. In this part, participants were also asked about what they used mobile phones for. In the part of intention and attitude about using the Internet and mobile phones for managing diabetes, participants were asked about services they required for managing their disease. The patients’ use of smartphone apps to manage their disease was assessed with seven questions: “For me, using smartphone apps for self-management can be a good idea, enjoyable, easy, exciting, interesting, helpful, and economical”. These questions are based on a 5-point Likert scale from very bad = 1 to very good = 5. Participants were also asked about their certainty about using smartphone apps for the disease management, and their intention to use apps in future was assessed by a question based on a 5-point Likert scale (from no intention = 1 to very high intention = 5).

### Data collection

The present study was approved by the ethics committee of Kashan University of Medical Sciences (IR.KAUMS.NUHEPM.REC.1396.21). To collect data, the researcher visited the diabetes clinic and specialists’ offices, and briefed the patients in the waiting room on the study objectives and how to complete the questionnaire. The questionnaires were distributed among the patients after they were reassured of confidentiality of data and signed written informed consents. The questionnaires were completed by the patients in researcher’s presence, so that the researcher could resolve any ambiguities. A total of 210 patients with T2DM attending the clinic and doctor’s offices were identified, of whom 176 were willing to complete the questionnaire, with a response rate of 83.8%. All 176 participants completed the questionnaires.

### Statistical analysis

Data were analyzed by using SPSS version 22.0. First, the percentage and frequency of demographic information, the use and intention to use mobile phones and the Internet by participants, and difficulties in self-management were determined. The overall attitude score was found by combining values of scales relating to attitude (7 questions) and finding their mean and standard deviation. The intention score was determined using one question. The body mass index (BMI) was calculated based on the details of weight and height reported by patients. The relationships between demographic information and attitude, and intention and confidence were assessed using Chi-square or Fisher’s exact test. *P* < 0.05 was considered as the level of significant.

## Results

Table 1 presents participants’ demographic and disease details. Of the 176 participating patients, 97 (55.1%) were women. Participants’ mean age was 53.18 years (SD = 15.5), the majority were did not complete high school (56.8%), and 152 (86.4%) lived in the city. Eighty-two patients had other chronic diseases (46.6%), of whom, 15 had hypertension, 8 had cardiac problems, and 4 had asthma, and the rest had other diseases including neurological, gastric, thyroid, kidney, and high blood lipids. Participants’ mean BMI was 28.42 kg/m^2^ (SD = 5.1).

**Table 1:** Participants’ demographic and disease details (*n* = 176)

Demographic details		Frequency	Percentage	Mean ± SD
Gender	Female	97	55.1	
	Male	79	44.9	
Age				53.18(±15.05)
Education level	Below high school diploma	100	56.8	
	High school diploma	40	22.7	
	Advanced diploma	7	4	
	Bachelor’s degree	20	11.4	
	Master’s degree and higher	9	5.1	
Occupation	Retired	21	11.9	
	Full-time employment	30	17	
	Part-time employment	8	4.5	
	Housewife	63	35.8	
	Unemployed	7	4	
	Other	47	26.7	
Place of residence	Urban	152	86.4	
	Rural	24	13.6	
Duration of the disease	Less than 6 months	35	19.9	
	6 months to one year	27	15.3	
	1 to 3 years	38	21.6	
	3 to 5 years	25	14.2	
	5 years and longer	50	28.4	
Other chronic diseases	Yes	82	46.6	
	No	92	52.3	
	Do not know	2	1.1	
BMI^a^				28.42 (±5.1)

^a^*BMI* Body mass index

Table 2 shows general findings about participants’ use of mobile phone and the Internet and their intention to use them for controlling their diabetes. Of the 176 participants, 167 (94.9%) had mobile phones, 109 (61.9%) had smartphones, and 143 (81.3%) had daily Internet access. Most of the participants spent more than two hours per day using mobile phones (80.1%) and the Internet (56.8%). Participants mostly used their mobile phones to contact friends (89.2%) and search for information (50.6%). They used their mobile phones the least for sending and receiving emails (9.1%), and shopping online (15.9%). Mobile phones and the Internet were used by most participants for planning their diet (96%), checking blood glucose and other relevant clinical parameters (90.9%), and contacting their physicians (87.5%).

**Table 2:** Frequency percentage of participants’ use and intention to use mobile phone and the Internet (*n* = 176)

Question		Number (percentage)		
		Yes	No	Do not know
Current technology use	Having a mobile phone	167 (94.9)	9 (5.1)	–
	Having a smartphone	109 (61.9)	67 (38.1)	–
	Having daily access to the Internet	143 (81.3)	33 (18.8)	–
Intention to use mobile phone and the Internet for diabetes control	Dietary planning	169 (96)	4 (2.3)	3 (1.7)
	Checking blood glucose and other parameters	160 (90.9)	4 (2.3)	12 (6.8)
	Contacting specialists	154 (87.5)	5 (2.8)	17 (9.7)
	Using text messages as a reminder for diabetes self-management	145 (82.4)	4 (2.3)	27 (15.3)
	Planning physical activity	143 (81.3)	9 (5.1)	24 (13.6)
	Contacting other healthcare providers (nutritionist and nurses)	100 (56.8)	20 (11.4)	56 (31.8)
	Contacting other patients with diabetes	45 (25.6)	63 (35.8)	68 (38.6)

Table 3 shows participants’ attitude and intention regarding the use of smartphone apps for diabetes self-management. Half of the participants stated that using apps to help manage diabetes can be interesting (54%) and useful (50%), 85 (48.3%) stated that they intended to use apps for diabetes control “much more” in future (48.3%), and 57 (32.4%) stated that they were 61 to 80% confident that they would use apps for controlling their diabetes.

**Table 3:** Mean and standard deviation of participants’ attitude and intention to use smartphone apps to control their diabetes (*n* = 176)

Question	Mean (out of 5)^a^
Patients’ attitude toward using apps for diabetes control	4.18 ± 0.68
Patients’ intention to use apps for diabetes control in the future	4.34 ± 0.75

^a^Attitudes & Intention rated 1–5

Table 4 shows the results relating to participants’ problems in diabetes self-management. The majority of the participants stated that their problems are mostly related to choosing an optimizing diet (81.8%), adequate physical activity (69.9%), and blood glucose diary (64.8%).

**Table 4:** Frequency percentage of participants’ problems in diabetes self-management

Diabetes self-management problems	Number (percentage)	
	Yes	No
Choosing the right diet	144 (81.8)	32 (18.2)
Doing adequate physical activity	123 (69.9)	53 (30.1)
Blood glucose diary	114 (64.8)	62 (35.2)
Contacting the physician	86 (48.9)	90 (51.1)
Not having clear and precise goals for diabetes management	77 (43.7)	99 (56.3)
Contacting other healthcare providers (nutritionists and nurses)	37 (21)	139 (79)
Contacting friends and family (for diabetes control)	11 (6.3)	165 (93.8)

Table 5 shows the correlation of participants’ demographic characteristics, attitude and intention to use smartphone apps for diabetes self-management and their Internet and mobile phone use. Compared to older participants, younger ones were significantly more interested (*P* < 0.001) in using apps, and had greater intention (*P* = 0.012) and higher confidence (P < 0.001) in using these tools in future. Moreover, younger participants spent significantly more time per day using their mobile phones (*P* < 0.001).

**Table 5:** Correlation between participants’ demographic characteristics, attitude and intention in relation to the use of smartphone apps for diabetes self-management

Demographic variables	Attitude	Intention	Confidence	Internet use	Mobile use
Age	−0.373^b^	−0.190^a^	−.470^b^	.290^b^	−.249^b^
Gender	0.092	0.066	.116	−.075	.030
Education	0.254^b^	0.083	.387^b^	.245^b^	.172^a^
Occupation	−0.157^a^	−0.044	−.229^b^	.074	−.053
Duration of diabetes	−0.161^a^	−0.060	−.203^b^	.225^b^	−.072
Place of residence^c^	–	–	–	–	–

^a^Correlation is significant at the 0.05 level (2-tailed)

^b^Correlation is significant at the 0.01 level (1-tailed)

^c^This comparison was not applicable

Participants’ gender had no significant relationship with attitude toward, intention to, and confidence in using smartphone apps and also daily use of the Internet and mobile phones. Patients with higher education levels had significantly better attitude toward (P < 0.001) and greater confidence (P < 0.001) in using smartphone apps, and also higher use of mobile phones (*P* = 0.022) and the Internet (*P* = 0.001). A significant difference was observed between patients’ occupation and confidence (P < 0.001) in using smartphone apps, such that the post hoc test results showed that participants in full-time employment had greater confidence in using apps. Patients with T2DM for a longer time had poorer attitude toward (*P* = 0.033) and less confidence in (*P* = 0.007) using apps compared to those having T2DMfor a shorter time. On the other hand, the Internet use significantly increased in those who had diabetes for a longer time (*P* = 0.003).

According to the results, 171 (97.2%) of the participants stated that they wished to receive help from other family members (namely spouse and children) if they were unable to use mobile phones for self-management purposes. Also, 125 (71%) wished to take part in future studies assessing the effect of using apps in diabetes control.

## Discussion

### Principal findings

The majority of the participants in the present study had mobile phone and access to the Internet. More than half of the participants spent two hours a day on average using their mobile phone and the Internet, and mobile phones were mostly used to contact friends and search for information. The majority of the participants intended to use mobile phones and the Internet for planning their diet, checking blood glucose and other clinical parameters, and contacting their doctors. Half of the participants stated that using smartphone apps can be interesting and help them manage their diabetes, and intended to use apps “very often” for diabetes control in future. About a third of the participants stated that they had confidence in using apps for diabetes control. The participants considered choosing the right diet, adequate physical exercise, and blood glucose diary as their main problems.

The results showed that a significant number of the participants had mobile phones and smartphones. Similarly, Boyle et al. [28] in New Zealand (2017), Humble et al. [30] in the UK (2015), and Dobson et al. [29] in Canada (2015) reported a significant number of patients with diabetes had access to mobile phones and smartphones. Also, a significant number of the participants in the present study had access to the Internet, and more than half of them used the Internet for two hours a day on average. Similarly, in a study by Dobson et al. [29] on 44 patients with T2DM, more than 90% of the participants had access to the Internet. The results of this study in a developing country compared to other studies in developed countries showed that there was no significant difference in terms of mobile phone and internet access. The results of the present and similar studies [28–30, 34] confirm that the increasing use of communication technologies (such as smartphones and the Internet) has created the opportunity to provide patients with chronic diseases with health information, especially self-management information.

The results showed that a significant number of the participants, who were mostly living in urban area, had mobile phones and smartphones. Similarly, in the studty by Hodge et al. [35], urban patients with T2DM in Australia had more use of Internet and mobile than rural residents. The lower Internet use associated with rural residence was explained by limited access to the Internet and to digital technology and differences in education and included personal limitations, such as a lack of skills, experience and familiarity in using online technologies [36, 37].

The majority of the participants stated their intention to use mobile phones and the Internet, especially for planning their diet, checking blood glucose, and contacting their doctors, which agrees with the findings of a study conducted in Canada [29]. More than half of the participants had a positive attitude toward, and the majority were confident in using apps for diabetes management, and half of the participant stated their intention to use apps in future for diabetes control. These results concur with those obtained by Conway et al. [38] in the UK (2015). Boyle et al. [28] cited that patients with diabetes expressed the following reasons for not using diabetes-related apps: not knowing such apps existed or their potential benefits, doctors did not recommend them to patients, patients did not feel confident without apps, and felt tired after using apps. According to Dobson et al. [29], the reasons for not using apps included the patients’ unawareness of the availability of such apps and lack of confidence in them. However, in a study by Jenkins et al. [39], 75% of patients with stroke had the intention to use mHealth interventions and 83.3% had confidence that this technology could be an effective tool for their communication with healthcare providers. Hofstede et al. (2014) [27] reported the main reason for a positive attitude of patients with asthma and chronic obstructive pulmonary disease toward using apps as being time-saving and user-friendly, and supporting planned care when required.

In the present study, the most common problems that patients with T2DM faced in self-management were choosing the right diet, inadequate exercise, and blood glucose diary. Similarly, Dobson et al. [29] reported choosing the right diet, inadequate exercise and following up blood glucose level as the most common problems of patients with T2DM. Given the importance of self-management in patients with T2DM and availability of diabetes-related apps with features for monitoring blood glucose, physical activity, and dietary management [40], several studies have investigated the effectiveness of such tools on diabetes-related outcomes [41–43]. These studies have shown that apps can improve adherence with activities relating to diabetes management such as regular intake of medication and insulin shots, blood glucose self-monitoring, diet, and physical activity.

In agreement with Dobson et al. study [29], the relationships of age and duration of T2DM with patients’ attitude and intention about using apps, and the use of mobile phones and the Internet were significant. Younger patients were more interested in using apps and had greater intention to and confidence in using them in future. Also, patients with diabetes for longer had poorer attitude toward and confidence in using apps. In the present study, patients with higher education levels had a better attitude toward and confidence in using apps, and used mobile phones and the Internet more often. These results agree with those obtained in studies by Song et al. [44] in the USA (2013) on pregnant women, and Jafari et al. [31] in Iran (2015) in patients with T2DM. A review study showed that most pregnant women with higher education considered using the Internet useful and reliable [45]. These results may be due to the fact that patients with higher education levels have greater reasoning and critical thinking skills compared to others, and therefore have better judgment and higher confidence in mHealth technologies.

To our knowledge, the present study is the first study in a developing country to investigate the attitude and intention of patients with T2DM about using the Internet and smartphone apps for diabetes self-management. The present study was conducted on a sample of 176 patients in a city, which may be considered as a limitation. Given that the majority of patients with T2DM were in older age group, with low education level, participants’ old age and poor education level can be considered another limitation.

According to the present study results, patients with a positive attitude toward and intention to use the Internet and smartphone apps for self-management were the younger ones, with higher education levels and a shorter period with diabetes. In the present study, the self-management problems of patients with T2DM were identified. It is recommended that diabetes self-management features of smartphone apps be designed according to the patients’ conditions and needs. As suggested in Dobson et al. study [29], future studies should identify barriers to and facilitators of using the Internet and mobile apps for diabetes self-management.

## Conclusion

Patients with T2DM in a developing country currently use mobile phones mostly for contacting friends and searching for information, and more than half of them have a positive attitude toward and high confidence in using smartphone apps to help manage their diabetes. The majority of patients with T2DM are inclined to use mobile phones and the Internet, especially to plan their diet, check blood glucose, and contact their doctors. The present study provides valuable information for designing and implementing interventions based on mHealth to promote self-management in T2DM.

## Supplementary information

**Additional file 1.** Final Questionnaire.

## Data Availability

The datasets used and/or analysed during the current study are available from the corresponding author on reasonable request.

## Abbreviations

apps: Applications
BMI: Body Mass Index
CVI: Content Validity Index
CVR: Content Validity Ratio
eHealth: electronic Health
mHealth: mobile Health
SPSS: Statistical Package for the Social Sciences
WHO: World Health Organization
