# Supplementary material for: Mobile phone usage in patients with type II diabetes and their intention to use it for self-management: a cross-sectional study in Iran
Source: BMC Med Inform Decis Mak. 2020 Feb 7;20:24. doi: 10.1186/s12911-020-1038-y (PMC7007646; doi:10.1186/s12911-020-1038-y)
Supplement: Supplementary file 1 — Additional file 1. Final Questionnaire. [file 12911_2020_1038_MOESM1_ESM.pdf]

Demographic Information

1- Gender: ☐ Female ☐ Male **2-** Age (year):… **3-** Place of residence: ☐ Urban ☐ Rural

4- Level of education: ☐ High school ☐ Diploma ☐ Associate degree ☐ undergraduate degree ☐ Postgraduate and higher

5- Employment status: ☐ Retired ☐ Working Full- Time ☐ Working Part Time ☐ Housewife ☐ Unemployed ☐ Others

Diabetes Management

6- Height (cm)… **7-** Weight (kg)…

8- How long have you been diagnosed with diabetes?
☐ Less than 6 months ☐ 6 months to 1 year ☐ 1 year to 3years ☐ 3 to 5 years ☐ Over 5 years ☐ Don't know/ Not Applicable

9- Do you have any other chronic health condition? ☐ Yes (name of disease…..) ☐ No ☐ Don't know

10- What area in your diabetes care do you find most difficult to manage or frustrating? (you can choose from several options)
☐ Making healthy diet choices ☐ Getting enough physical activity ☐ Communicating with my physician
☐ Communicating with other Health care Provider, such as dieticians, nurses, etc. ☐ Communicating with friends and family
☐ Taking and keeping track of my blood glucose levels ☐ Not having clear and concrete goals for your diabetes ☐ others............

Use of smartphone/cell phone and internet

11- Do you own a cellphone? ☐ Yes ☐ No

12- Do you own a smart phone ?(such as Android) ☐ Yes ☐ No

13- Do you have daily access to the internet at home? ☐ Yes ☐ No

14- On average, how many hours a day do you spent on the internet?
☐ Less than one hour ☐ Between 1 to 2 hours ☐ Between 2-3 hours ☐ Over 3 hours ☐ Don't know

15- On average, how many hours a day do you spent on your smartphone or cell phone?
☐ Less than one hour ☐ Between 1 to 2 hours ☐ Between 2-3 hours ☐ Over 3 hours ☐ Don't know

16- What activities do you typically use your smartphone (tablet) for? (you can choose from several options)
☐ E-mailing ☐ Communicating with friends ☐ Social media such as telegram ☐ Reading the news or books
☐ Researching Information ☐ Watching movie ☐ internet shopping ☐ others...........

Your attitude to Diabetes self-management with using internet and smartphone application

17- If you were to use an internet or smartphone application to help manage your diabetes, select what management services you would be interested in:

	Don't know	No	Yes
Dietary planning	☐	☐	☐
Physical activity planning	☐	☐	☐
Text Messaging Monitoring and/ or Reminders	☐	☐	☐
Glucose Reading and Tracking options	☐	☐	☐
Communication with other people with Type2 Diabetes	☐	☐	☐
Communication with diabetes clinicians	☐	☐	☐
Communicating with other Health care Provider, such as dieticians, nurses, etc.	☐	☐	☐

18- For me, using a specially developed smart phone application to assist with self-management would be:
☐ Very bad idea ☐ Bad idea ☐ Neither a bad or good idea ☐ Good idea ☐ Very good idea

19- For me, using a specially developed smart phone application to assist with self-management would be:
☐ Extremely unenjoyable ☐ Unenjoyable ☐ Neither unenjoyable or Enjoyable ☐ Enjoyable ☐ Very Enjoyable

20- For me, using a specially developed smart phone application to assist with self-management would be:
☐ Very Frightening ☐ Frightening ☐ Neither Frightening or comforting ☐ Comforting ☐ Very comforting

21- For me, using a specially developed smart phone application to assist with self-management would be:
☐ Very Boring ☐ Boring ☐ Neither Boring or Exciting ☐ Exciting ☐ Very Exciting

22- For me, using a specially developed smart phone application to assist with self-management would be:
☐ Very Dull ☐ Dull ☐ Neither Dull or Interesting ☐ Interesting ☐ Very Interesting

23- For me, using a specially developed smart phone application to assist with self-management would be:
☐ Very Unhelpful ☐ Unhelpful ☐ Neither Unhelpful or helpful ☐ helpful ☐ Very helpful

24- For me, using a specially developed smart phone application to assist with self-management would be:
☐ Very time Consuming ☐ Time Consuming ☐ Neither time Consuming or time saving ☐ time saving ☐ Very time saving

25- I feel ….% confident that I could use a smart phone application to help me with my diabetes.
☐ 0% –20% ☐ 21% - 40% ☐ 41% – 60% ☐ 61% – 80% ☐ 81% – 100%

26- If you were unable to use a smart phone would you want a spouse/friend/ family member to help you manage your diabetes with a smart phone?
☐ Yes ☐ No

27-I intend to use a smart phone application for helping me with my diabetes management in the future.
☐ Not at all ☐ Very Little ☐ Somewhat ☐ A little ☐ Very Much

General comments and additional contact Request

28- If you have any additional comments about using smart phone application manage diabetes, please state so here: ....
.

29- Would you be willing to be contacted to further discuss your smart phone use in future? ☐ Yes ☐ No
(Please provide your name, phone number……….)

30-Would you be willing to be contacted to participate in any other study to management diabetes with use of smart phone?
☐ Yes ☐ No (please provide your name, phone number…….)
